# Transcriptomic analysis of nonylphenol effect on *Saccharomyces cerevisiae*

**DOI:** 10.7717/peerj.10794

**Published:** 2021-02-11

**Authors:** Ceyhun Bereketoglu, Gozde Nacar, Tugba Sari, Bulent Mertoglu, Ajay Pradhan

**Affiliations:** 1 Department of Biomedical Engineering, Faculty of Engineering and Natural Sciences, Iskenderun Technical University, Hatay, Turkey; 2 Department of Bioengineering, Faculty of Engineering, Marmara University, Istanbul, Turkey; 3 Biology, The Life Science Center, School of Science and Technology, Örebro University, Örebro, Sweden

**Keywords:** Nonylphenol, RNA-seq, *Saccharomyces cerevisiae*, OXPHOS, Cell cycle process

## Abstract

Nonylphenol (NP) is a bioaccumulative environmental estrogen that is widely used as a nonionic surfactant. We have previously examined short-term effects of NP on yeast cells using microarray technology. In the present study, we investigated the adaptive response of *Saccharomyces cerevisiae* BY4742 cells to NP exposure by analyzing genome-wide transcriptional profiles using RNA-sequencing. We used 2 mg/L NP concentration for 40 days of exposure. Gene expression analysis showed that a total of 948 genes were differentially expressed. Of these, 834 genes were downregulated, while 114 genes were significantly upregulated. GO enrichment analysis revealed that 369 GO terms were significantly affected by NP exposure. Further analysis showed that many of the differentially expressed genes were associated with oxidative phosphorylation, iron and copper acquisition, autophagy, pleiotropic drug resistance and cell cycle progression related processes such as DNA and mismatch repair, chromosome segregation, spindle checkpoint activity, and kinetochore organization. Overall, these results provide considerable information and a comprehensive understanding of the adaptive response to NP exposure at the gene expression level.

## Introduction

Nonylphenol ethoxylates (NPEs) are cost-effective non-ionic surfactants that are widely used in household, commercial, industrial and agricultural applications. It is used as detergents, emulsifiers, demulsifiers, solubilizers, antistatic agents, wetting and dispersing agents, cosmetics, plastics and paints (De la Parra-Guerra & Olivero-Verbel, 2020; Li, Jin & Snyder, 2018; Noorimotlagh et al., 2018; Soares et al., 2008). NPEs are readily biodegradable due to the presence of ethoxy groups. The stepwise loss of these groups during the degradation process leads to the formation of nonylphenol (NP) (De Bruin et al., 2019; Li, Jin & Snyder, 2018). Anaerobic conditions are favored for NP formation; however, some studies have indicated that oxic conditions can also drive the reaction (Careghini et al., 2015; Micic & Hofmann, 2009). NP has high hydrophobicity and lipophilic characteristics (log K_ow_ between 3.8 and 4.8) with lower solubility and mobility (Duan et al., 2019; Mattana et al., 2019). Half-life of NP varies between 0.9 and 13.2 days under oxic conditions. The first-order half-life of NP isomers could be >200 days under strongly reduced conditions (Careghini et al., 2015). Hence, NP is a relatively stable chemical and accumulate in different environmental matrices including ground and surface waters, drinking waters, soils, sediments, atmosphere and air (Careghini et al., 2015; De Bruin et al., 2019; Forte et al., 2016; Yang et al., 2020).

Nonylphenol mimics the natural estrogen 17β-estradiol and thereby alters estrogen function at different development and reproductive stages of several model systems (Noorimotlagha et al., 2020; Rachon, 2015). NP has been detected in human adipose tissue, placenta (5.4–54.4 ng NP/g ), maternal blood, breast milk (0.07–57.3 ng NP/ml), urine (0.1–3.7 µg NP/L), plasma samples (53.21 ± 49.74 ng NP/g in housekeeping workers) and hair (4478 ng NP/g) (Chen, Chang & Minatoya, 2020; Chen et al., 2005; Forte et al., 2016; Huang et al., 2014; Nehring, Staniszewska & Falkowska, 2017). The NP concentration in breast milk from Turkish mothers was found to be up to 47 ng/ml (Sise & Uguz, 2017), while 0.1–100 µg NP/kg fresh weight (FW) has been detected in various food content (Acir & Guenther, 2018). In addition, 0.067–0.52 µg/kg of body weight (BW) has been estimated as the average daily uptake value of NP and the tolerable daily intake for NP has been proposed as 5 µg/kg BW/day by The Danish Institute of Safety and Toxicology (Chen, Chang & Minatoya, 2020).

Toxicogenomics (genomics, epigenomics, transcriptomics, proteomics and metabolomics) are implemented to understand the molecular mechanisms of compounds or pollutants (Auerbach, 2017; Dos Santos & Sa-Correia, 2015). RNA sequencing (RNA-seq) is an advanced toxicogenomic tool used for generating whole-genome transcriptomic data by high-throughput sequencing technologies. In recent years, it has started to replace hybridization-based microarray platforms because of its advantages such as highly reproducibility, detection of low abundance transcripts, identification of genetic variants, relatively little technical variation and non-reliance on probe selection (Reed et al., 2019; Zhao et al., 2014).

*Saccharomyces cerevisiae (S. cerevisiae)*, a budding yeast, is a eukaryotic model organism that is used in various studies to investigate aging, cell cycle, neurodegenerative diseases, regulation of gene expression and many different biological processes (Karathia et al., 2011). *S. cerevisiae* has simple growth conditions, rapid reproductive rates, and can be manipulated easily (Dos Santos & Sa-Correia, 2015). Moreover, the annotation and characterization of this organism were performed (Goffeau et al., 1996), and the data uploaded to different publicly available databases (Dos Santos & Sa-Correia, 2015). Besides, *S. cerevisiae* has several structures and functions which are similar with higher eukaryotes including humans. Around 17% of yeast genes are members of orthologous gene families associated with human disease. The mammalian homologs for the majority of these genes are functional in yeast and complement yeast deletion mutants. Besides, for most of the genes, protein amino acid sequences and protein functions in yeast have been conserved (Botstein & Fink, 2011; Dos Santos et al., 2012; Heinicke et al., 2007). Hence, this organism could potentially be used to reveal the underlying molecular mechanisms of toxicity of pollutants and extrapolate the data in higher eukaryotes (Duina, Miller & Keeney, 2014; Nomura et al., 2013).

Several studies have investigated the short- and long-term effects of NP on different species to reveal its toxicity at the gene level (Duan et al., 2017; Forte et al., 2016; Jin et al., 2010; Kazemi et al., 2016; Robertson & McCormick, 2012; Shelley et al., 2012; Won, Woo & Yum, 2014; Xu et al., 2020). However, most of these studies have focused on only specific genes rather than determining the impact on the whole genome. We have previously determined the short-term effects (acute toxicity) of NP on *S. cerevisiae* using microarray platform (Bereketoglu et al., 2017b). We found that exposure to NP resulted in substantial changes in the expression of genes involved in oxidative phosphorylation, cell wall biogenesis, ribosomal biogenesis, RNA processing, and genes encoding heat shock proteins and ubiquitin-conjugating enzymes. In this follow-up study, we examined the adaptive response of yeast cells towards NP at environmentally relevant concentration. The transcript profile revealed that NP affects different signaling pathways including, cell cycle, iron and copper metabolism, mitochondrial oxidative phosphorylation (OXPHOS) and autophagy.

## Materials and Methods

### Strain, growth conditions and NP exposure

*Saccharomyces cerevisiae* BY4742 (Matα; his3Δ1; leu2Δ0; lys2Δ0; ura3Δ0) was obtained from the Euroscarf collection (Frankfurt, Germany). To obtain single colonies, prepared yeast extract-peptone-glucose (YPD) agar (2% (w/v) D-glucose, 2% (w/v) peptone, 1% (w/v) yeast extract, 1% (w/v) agar) plates were spread with stock yeast cells using sterile loop and incubated at 30 °C for 48 h. *S. cerevisea* cells were cultured at 30 °C in a shaking incubator. The shaking speed was maintained at 180 rpm. To prepare the starting culture, 25 mL of YPD medium (2% (w/v) D-glucose, 2% (w/v) peptone, 1% (w/v) yeast extract) was taken and a single yeast colony was inoculated from an overnight cultured yeast plate. From the starting culture, 1% of the culture was taken and inoculated in 100 mL YPD medium and cultured.

Nonylphenol stock solution was prepared in ethanol, and then the first round of the exposure was initiated by adding NP to the cultures to reach final concentrations (0.1, 1 and 2 mg/L NP) at time 0 h. These concentrations were chosen based on previous study (Bereketoglu et al., 2017b). The final concentration of ethanol in the medium was maintained at 1% (vol/vol), which did not affect growth. Ethanol, 1% (vol/vol) was used as vehicle control. At the end of 22 hr of exposure, 1 mL sample was taken from each of the cultures, centrifuged, and the pelleted cells were suspended in 1 mL of liquid YPD medium. This process was repeated three times to remove possible NP residuals. After the washing procedure, suspended cells were transferred to the new 100 mL YPD in 500 mL flasks and NP was added to the cultures to reach the above mentioned final concentrations of NP, and the second round of the exposure was initiated. The exposure was performed for 40 rounds by repeating the above steps.

To determine the inhibition/mortality rates following NP exposure, cell viability analysis was performed by vital staining with methylene blue solution (0.01% methylene blue, 2% sodium citrate), as described previously (Bereketoglu et al., 2017b). Briefly, aliquots of yeast cultures exposed to NP for 8 h were taken and stained with methylene blue solution. The number of dead (stained blue) and live (unstained) cells were determined microscopically within 10 min of staining. The growth curve for the selected chemical concentrations was determined by monitoring optical density at 600 nm. Briefly, optical density measurements were conducted every 4 h with a spectrophotometer (PhotoLab 7100 VIS, WTW, Germany). Three independent experiments were performed for each concentration. Finally, samples were harvested and snap freezed in liquid nitrogen, and stored at −80 °C until the RNA isolation.

### RNA isolation and RNA-seq analysis

RNA isolation was performed using the mechanical lysis protocol provided with the RNeasy Mini Kit (Cat. no.: 74104; Qiagen, Venlo, Netherlands). RNA quality and quantity were analyzed using a NanoDrop ND-100 UV-Vis spectrophotometer (Thermo Fisher Scientific Inc., Waltham, MA, USA). RNA integrity (RIN) was determined with an Agilent Bioanalyzer 2100 (Agilent Technologies, Santa Clara, CA, USA) device using an RNA 6000 Nano Assay kit (Agilent Technologies, Santa Clara, CA, USA), and only those samples with RIN values between 6.5 and 10 were used for further analysis. Triplicate RNA samples with the best quality were selected for transcriptional analysis.

RNA-Seq was performed by BGI Genomics Organization, Ltd (Hong Kong, China). Libraries for RNA-Seq were prepared using the following procedure. The poly-A mRNA was purified and fragmented into small pieces. The RNA was converted into first strand cDNA and then second strand was synthesized using DNA polymerase I and RNase H. This was followed by addition of single Adennine base and attachment of adapter sequence. The modified fragments were purified and amplified using PCR. The PCR yield was quantified by Qubit and pooled samples were pooled to make a single strand DNA circle (ssDNA circle), to get the final library. DNA nanoballs (DNBs) were generated using the ssDNA circle. The DNBs were then read through on the BGISEQ-500 platform and stepwise sequencing using Combinational Probe-Anchor Synthesis Sequencing Method was performed. The RNA-Seq data is submitted to Gene Expression Omnibus (GEO) under accession number GSE158124.

### Data analysis

RNA-Seq data was analyzed using Partek Genomic Flow software (Partek, St. Louis, MO, USA). The raw data files were first analyzed for sequence quality using pre-alignment QA/QC. The reads were then aligned to the *S. cerevisiae* genome (R64-1-1) using STAR 2.5.3a aligner index (Dobin et al., 2013). Genome assembly and annotation files were obtained from Ensemble genome browser. The generated counts were normalized using counts per million (CPM) method and the differentially regulated genes were identified using differential gene expression (GSA) algorithm (Law et al., 2014). Then, gene ontology (GO) enrichment anaylsis was performed to determine GO terms. For pathway analysis KEGG pathway database was used (Kanehisa & Goto, 2000). False discovery rate (FDR) was set to 0.05, *p* value to 0.05 and fold change +/− 1.5 fold. To summarize the gene expression analysis, principal component analysis (PCA) was performed. An average linkage hierarchical cluster analysis was performed to compare the difference in the global gene expression profiles of the treated and control samples. To generate a functional relationship network structure, the enriched GO terms were entered into the Reduce and Visualize Gene Ontology (REViGO; http://revigo.irb.hr/) and summarized by removing redundant GO terms (Supek et al., 2011).

### Validation of RNA-seq data using quantitative real-time PCR

To validate the transcriptomic data, quantitative real-time PCR (qPCR) analysis was performed. Five genes were selected for qPCR analysis. The cDNA samples were synthesized from equivalent amounts of the total RNA samples used in the RNA-Seq analysis, using a qScript cDNA Synthesis Kit (Quanta, Gaithersburg, MD, USA), according to the manufacturer’s instructions. Then, qRT-PCR was carried out with a Light-Cycler device using a LightCycler FastStart DNA Master SYBR Green I Kit (Roche Molecular Biochemicals, Mannheim, Germany), according to the manufacturer’s instructions. Primer sequences were either obtained from previously published studies (Bereketoglu et al., 2017b; Bisquert, Muñiz-Calvo & Guillamón, 2018; Ye et al., 2006) (Table S1). Thermocycling conditions were as follows: 1 cycle of initial denaturation at 95 °C for 10 min, followed by 45 quantification cycles of denaturation at 95 °C for 10 s, primer annealing at primer-specific temperatures for 10 s, and primer extension at 72 °C for 25 s. The fold changes were calculated according to the ΔΔCt method, as described by Schmittgen & Livak (2008). Actin (*ACT1*) was used to normalize gene expression. All exposed and control samples were analyzed in triplicate.

## Results

### Inhibition of *S. cerevisiae* BY4742 growth by treatment with NP

**Figure 1:**
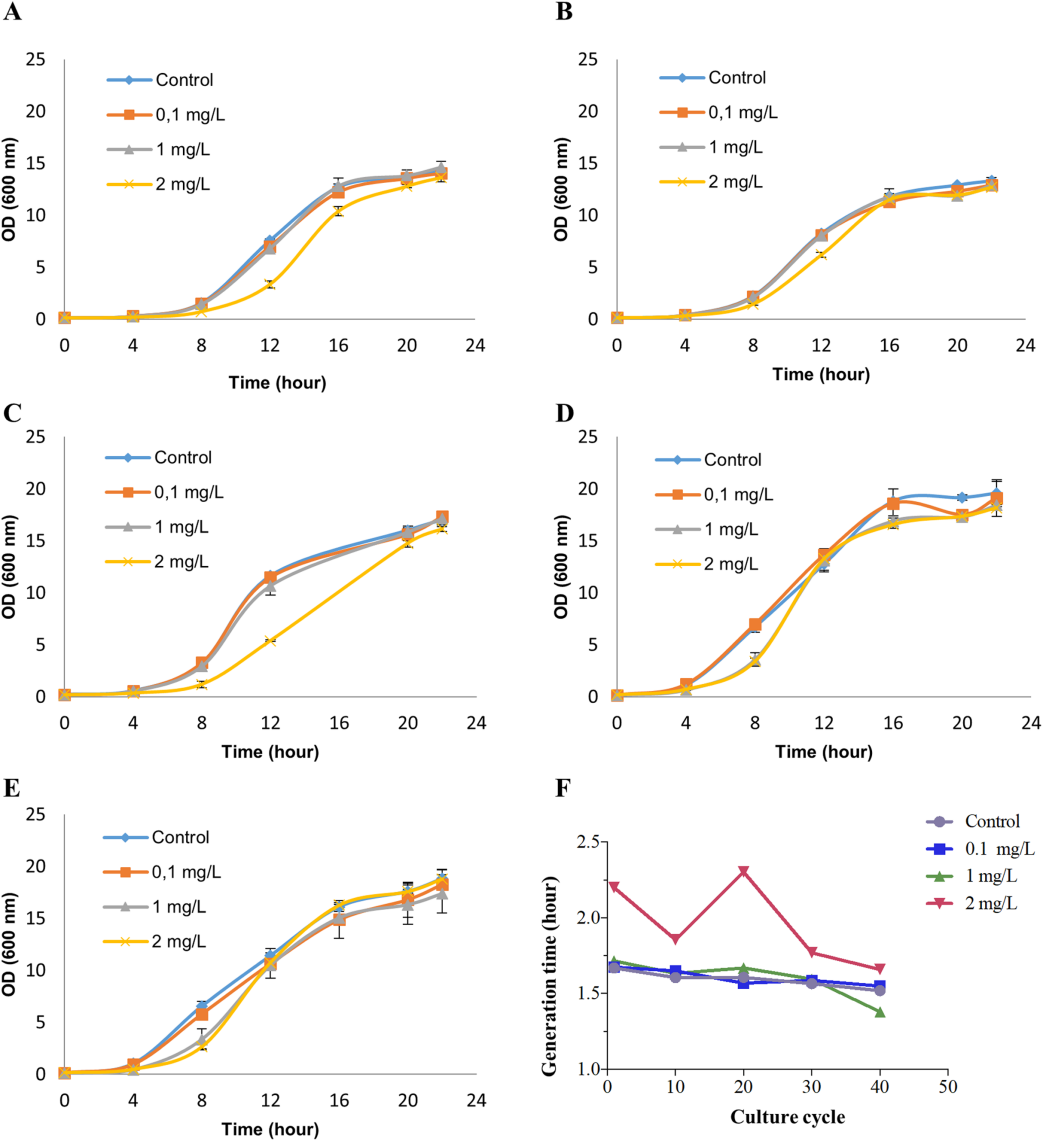
Growth patterns of *Saccharomyces cerevisiae* strain BY4742 upon exposure to various concentrations of nonylphenol (NP). Cells were exposed to 0.1, 1 and 2 mg/L concentrations of NP and the growth was recorded by measuring optical density at 600 nm. (A) The 1st round, (B) the 10th round, (C) the 20th round, (D) the 30th round and (E) the 40th round of the exposure period. (F) Generation time was determined for the growth data.

*Saccharomyces cerevisiae* BY4742 cultures were treated with 0 (control), 0.1, 1 and 2 mg/L NP (in ethanol) for 40 growth cycles (from 0 to 24 h in the growth curve for each growth cycle) by refreshing YPD culture and NP every cycle. These exposure concentrations were chosen based on our previous study (Bereketoglu et al., 2017b). To determine the appropriate concentrations of NP for RNA-seq analysis, we measured cell viability by methylene blue staining (Supplemental Material 1). No cell death was observed at any of the above concentrations, while at 2 mg/L NP exposure dose the growth was inhibited at the earlier growth cycles (Figs. 1A–1C). Although cells exposed to 2 mg/L NP showed an inhibition in the growth at the earlier growth cycles, they adapted to the treatment and had a similar growth pattern as control (Figs. 1D and 1E). We, therefore, established 2 mg/L NP as an appropriate concentration to investigate the transcription profiles of yeast. Finally, the growth was analyzed by measuring optical density at 600 nm which showed that 2 mg/L NP resulted in a distinct growth pattern compared with other doses and control (Fig. 1). We further calculated the generation time at log phase and found that 2 mg/L NP exposure group had a higher generation time compared to other exposure groups and the control at early grotwh cycles (Fig. 1F). However, at later growth cycles, yeast cells in this group showed an adaptive response to NP exposure and generation time decreased to the control level (Fig. 1F).

### Gene expression analyses of yeast cells exposed to 2 mg/L of NP

**Figure 2:**
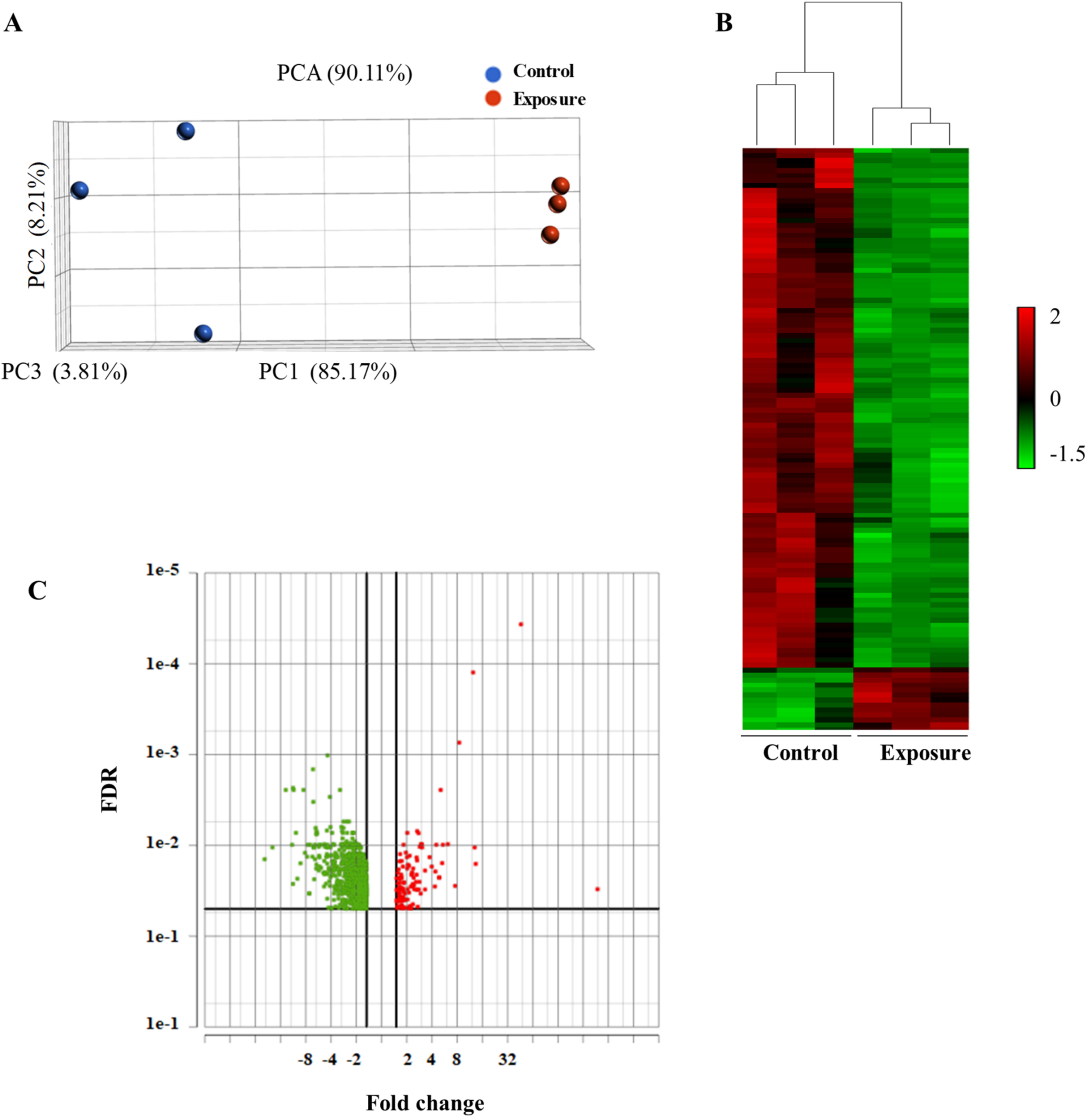
Principal component analysis (PCA) and hierarchical cluster analysis of gene expression data between the NP exposed and the control yeast cells. (A) The PCA score plot showing the distribution of NP and control based on gene responses. (B) The cluster analysis displaying gene expression changes in the yeast cells of NP exposed and control. (C) Volcano plot of the upregulated and downregulated genes. The volcano plot shows the upregulated and downregulated DEGs in yeast cells in response to NP. For each plot, the *x*-axis represents the fold change (FC). Genes with false discovery rate (FDR) and *p* value of less than 0.05 were assigned as differentially expressed.

To analyze the global gene expression following NP treatment, mRNA was isolated from three individual biological replicates, and RNA-seq was performed. We performed PCA to summarize gene expression profiles. The principal component 1 explained 85.17% of the variation and the second component 8.21%. PCA showed that the model can be used to differentiate the treated and the control samples (Fig. 2A). We then performed an average linkage hierarchical cluster analysis to compare the difference in the global gene expression of the NP-treated samples with the control (Fig. 2B). All the treated samples were clustered and separated from the control samples, suggesting that there is a distinct difference in gene expression between control and treated samples.

Differentially expressed genes (DEGs) following 40 growth cycles exposure to 2 mg/L NP were determined. Genes were considered to be differentially expressed with an adjusted *p* value of less than 0.05 and applying a 1.5-fold cutoff. RNA-seq analysis revealed a total of 948 differentially expressed genes in yeast exposed to NP compared with the control. Of these, 834 were downregulated (Fig. 2C, green dots) and 114 were upregulated (Fig. 2C, red dots). A complete list of these genes and their expression levels can be found in Supplemental Material 2.

### GO enrichment and pathway analyses of DEGs

**Figure 3:**
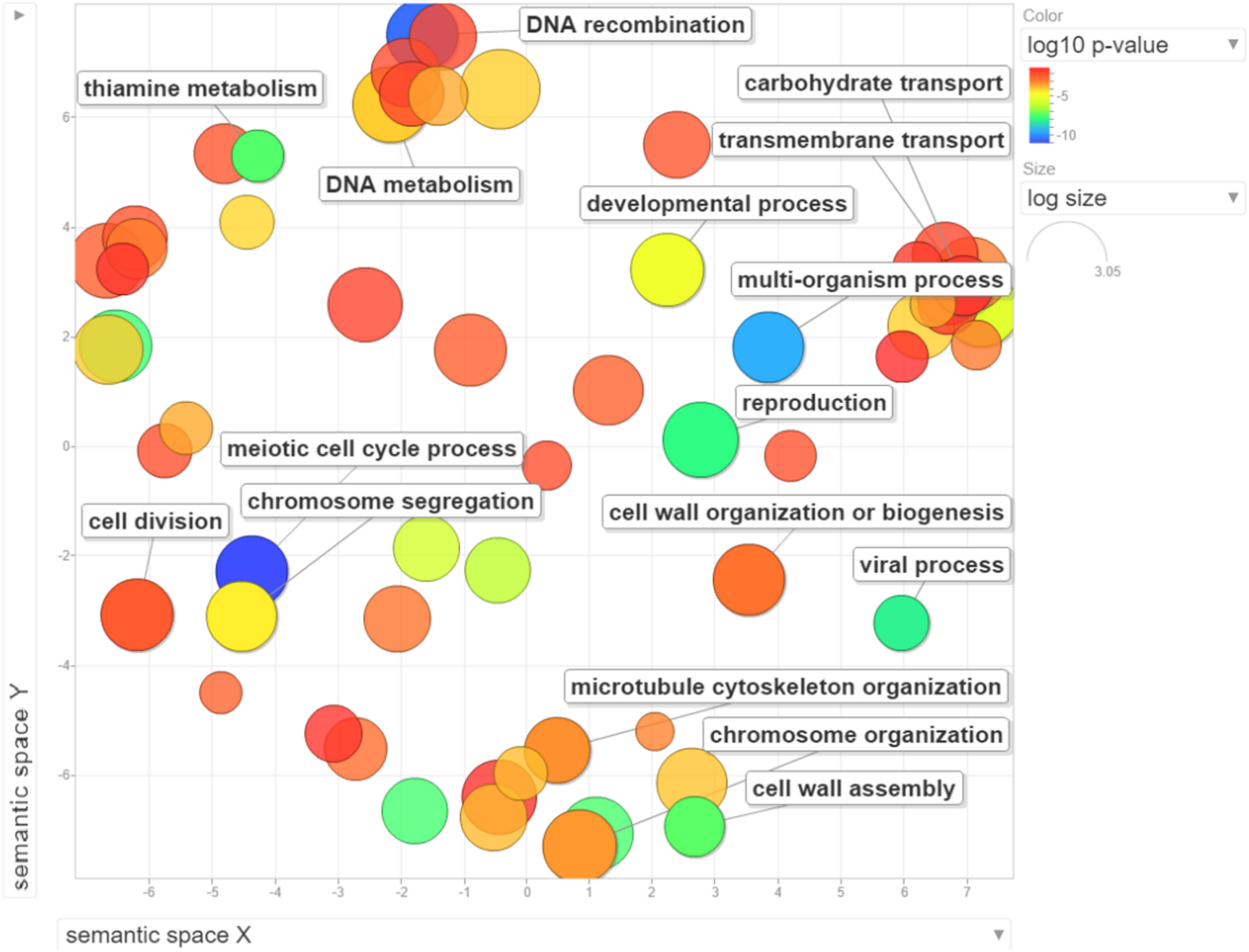
Visualization of GO enrichment analysis for differentially expressed genes under NP treatment. Circle size represents the frequency of the GO term, while color indicates the log10 *p*-value. Red higher; blue lower.

**Figure 4:**
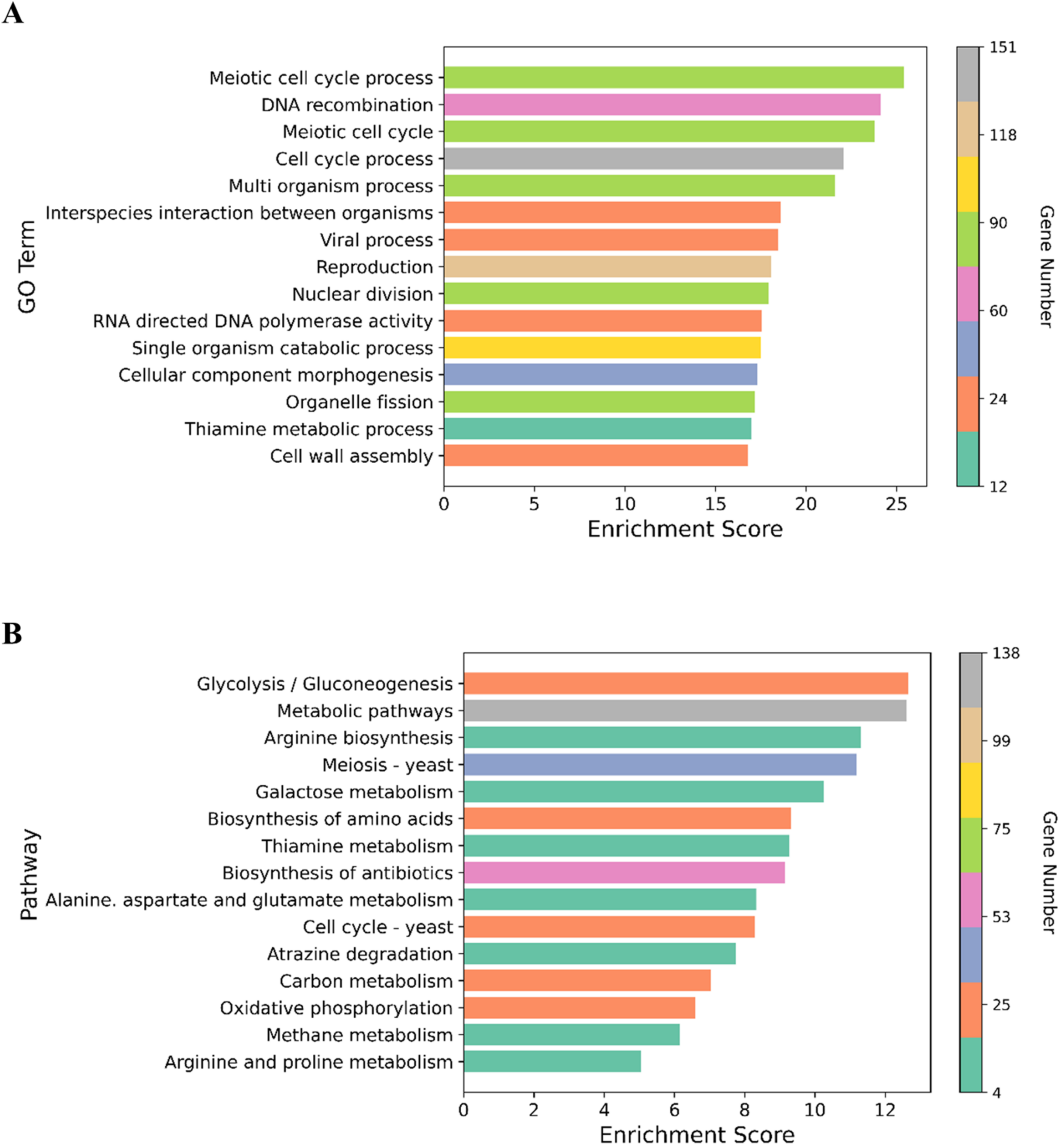
Graphical representation of the enriched GO terms and pathways. (A) The top 15 enriched GO terms. (B) The top 15 enriched pathways. Color bar indicates the number of the genes in the GO terms and pathways. *X*-axis represents enrichment score in the GO terms and pathways.

We performed GO enrichment analysis to determine the DEGs in the NP-treated group compared with the control. GO enrichment analysis revealed that 369 GO terms were significantly affected (For the whole list see Supplemental Material 3). Many of the DEGs were associated with cell cycle processes (meiotic cell cycle process, cell division, chromosome segregation and chromosome organization), DNA associated processes including DNA recombination and DNA metabolism, cell wall assembly, cell wall organization or biogenesis, and transport processes such as carbohydrate and transmembrane transports (Fig. 3). The top 15 enriched GO terms are shown in Fig. 4A. To identify the significantly affected pathways, KEGG pathway enrichment analysis was applied. DEGs were significantly clustered in 25 pathways (For the whole list see Supplemental Material 4). Moreover, several genes were associated with KEGG pathways including glycolysis/gluconeogenesis, metabolic pathways, arginine biosynthesis, meiosis-yeast and galactose metabolism. The top 15 enriched KEGG pathways are shown in Fig. 4B. Of special interest, cell cycle—yeast, oxidative phosphorylation and mismatch repair pathways were also significantly affected (Supplemental Material 4; Each of these functions will be discussed in more depth in subsequent sections).

### qPCR validation of RNA-seq transcriptome analysis

**Figure 5:**
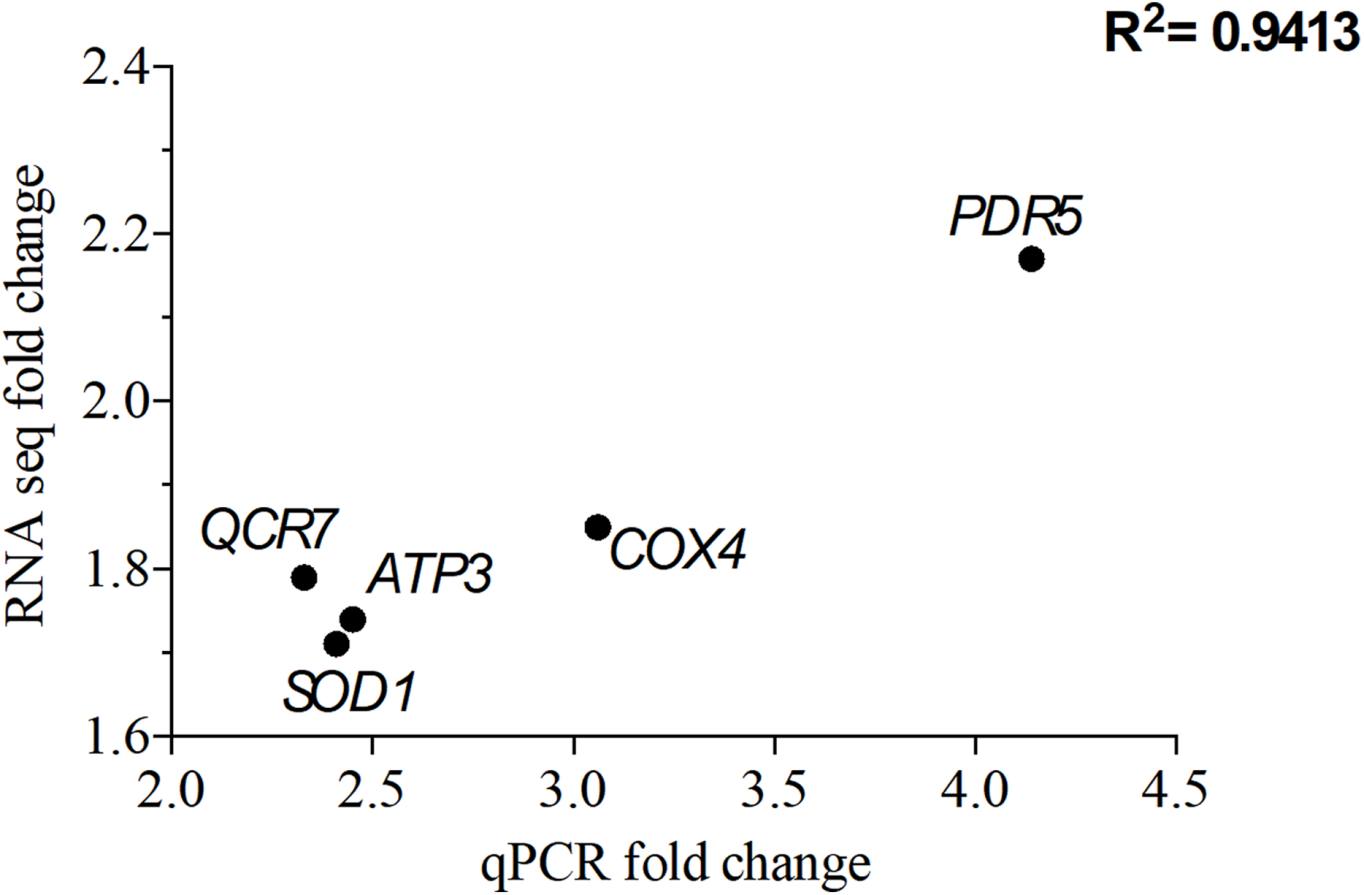
Correlation of gene expression from quantitative real-time RT-PCR and RNA-seq data. The differentially expressed genes (DEGs) were chosen from the significantly enriched Gene Ontology (GO) terms to validate RNA-seq data quantitative using real-time PCR. Y-axis represents RNA-seq fold change of the chosen genes, while *x*-axis represents quantitative real-time RT-PCR fold change.

To validate the transcriptomic data, qPCR was perforemd using the same RNA samples used for RNA seq. A total of five genes that were overexpressed upon exposure to 2 mg/L NP were chosen. Of these five genes, *QCR7*, *COX4* and *ATP3* are involved in the OXPHOS system, while *PDR5* (ABC superfamily) and *SOD1* (superoxide dismutase) are stress response genes. The correlation coefficient, *R*^2^ of 0.94 for the analyzed five genes suggests that the RNA seq data is consistent with the qPCR data (Fig. 5).

## Discussion

### Exposure to NP affected growth pattern of yeast cells

Environmental pollutants including NP are continuously released into and accumulate in natural environments. Hence, they have become a potential threat to organisms and ecosystems (Mao et al., 2012). It is of great importance to provide information about their mode of actions to regulate their use in the future. Previously, we showed that the short-term exposure to 1 and 5 mg/L of NP inhibited the growth and resulted in substantial changes in the transcriptional profile of model organism *S. cerevisiae* BY4742 (Bereketoglu et al., 2017b). However, organisms may show distinct acute and chronic exposure profile. Hence, in the present study, we examined the adaptive response of *S. cerevisiae* to NP exposure in. The data showed that 2 mg/L NP resulted in inhibition of yeast growth at the earlier growth cycles, while the cells adapted in the later stages (Fig. 1F). Lower concentrations of NP did not show any inhibition in the growth. However, in our previous study, short-term exposure to even 1 mg/L NP showed a growth inhibition in cells (Bereketoglu et al., 2017b). These differences could be due to the difference in exposure time. In the present study, we started to expose yeast cells to NP at time 0 h whereas in the previous study, treatment was performed at mid-exponential phase. Based on the inhibition rates and growth patterns, 2 mg/L NP was chosen as an appropriate concentration for further analysis.

### RNA-seq analysis showed a substantial change in gene expression, clustering in GO terms and metabolic pathways

Principal component analysis analysis showed that yeast cells treated with NP were distinct from those of the controls (Fig. 2A). We observed a total of 948 DEGs in yeast exposed to NP. Of these, 834 were downregulated and 114 were upregulated (Fig. 2C). The number of DEGs in the present study was significantly higher than that of observed in our previous study (Bereketoglu et al., 2017b). In the previous study, after 120 and 180 min exposure, 1 mg/L NP resulted in 187 (63 downregulated, 124 upregulated) and 103 DEGs (56 downregulated, 47 upregulated), while 5 mg/L NP showed 678 (168 repressed, 510 induced) and 688 DEGs (215 repressed, 473 induced), respectively (Bereketoglu et al., 2017b). This finding indicates that exposure to NP causes severe transcriptomic changes compared to short-term exposure. We further compared the number of common genes between the two studies. We determined 17 and 20 common genes with 1 mg/L and 5 mg/L NP exposures at 120 and 180 min exposure, respectively. Only seven genes were common with two exposure time-points (Fig. S1). Of these, *PDR5* and *GRE2* are involved in stress response, while *RSB1* and *TPO2* are transporter genes. Besides, *FRE5* and *GAC1* are involved in iron and glycogen metabolisms, respectively. Several common and distinct biological processes and metabolic pathways were also determined between the present study and previous study (Bereketoglu et al., 2017b). This suggests that yeast cells may respond to short- and adaptive response exposure to NP via common and unique processes/pathways.

### NP treatment resulted in increased expression of genes involved in OXPHOS and encoding mitochondrial ribosomal proteins

Mitochondria are the main energy producers for cell growth, differentiation and development. The majority of the cellular energy in the form of ATP in eukaryotic cells is produced by the key metabolic process called OXPHOS. The OXPHOS system comprises of five enzyme complexes (complex I to complex V) and two electron carriers (quinone-coenzyme Q and cytochrome-C) that helps to generate proton gradient for energy production (Fernández-Vizarra, Tiranti & Zeviani, 2009; Reinecke, Smeitink & Van der Westhuizen, 2009; Sylvester et al., 2004). Besides, mitochondria is also involved in regulating cell survival and death signaling. Several neurological problems, including Parkinson’s disease, Alzheimer’s disease and Huntington’s disease, are associated with mitochondrial functions (Diaz, 2010; Manczak et al., 2004). The OXPHOS system was significantly affected as several genes involved in this system was upregulated in response to 2 mg/L NP. In the present study, one gene (*SDH7*) associated with mitochondrial complex II (i.e., succinate dehydrogenase (SDH)); three genes (*COR1*, *QCR6* and *QCR7*) associated with mitochondrial complex III (i.e., ubiquinol-cytochrome c reductase complex); four genes (*COX1*, *COX4*, *COX5A* and *COX8*) associated with mitochondrial respiratory chain complex IV, called as cytochrome oxidase (COX); and nine genes (*ATP1*, *ATP2*, *ATP3*, *ATP4*, *ATP5*, *ATP7*, *ATP11*, *ATP20* and *OLI1*) associated with complex V, known as F0F1-ATP synthase were overexpressed. Moreover, cytochrome c1 (*CYT1*)—component of the mitochondrial respiratory chain, cytochrome c heme lyase (*CYC3*)—that play a role in attaching heme to apo-cytochrome c in mitochondrial intermembrane space, PETite (*PET9*)—major ADP/ATP carrier of the mitochondrial inner membrane, and NADH dehydrogenase, external (*NDE1*)—mitochondrial external NADH dehydrogenase subunits were also significantly induced in response to NP exposure. These findings are in contrast to our previous observations on the acute toxicity of NP (Bereketoglu et al., 2017b) and bisphenol A (BPA) (Bereketoglu et al., 2017a). Specifically, acute toxicity of NP resulted in reduced expression of many of the above and several other genes involved the OXPHOS system (Bereketoglu et al., 2017b). Besides, several studies have shown that mutations and defects in various subunits of OXPHOS may cause various neuromuscular disorders in humans (Kucharczyk et al., 2009) and OXPHOS genes are upregulated in a certain type of cancers reviewed in Ashton et al. (2018). In another study, it has been revealed that complexes III and IV showed increased mRNA expressions in Alzheimer’s disease patients, suggesting a great demand on energy production. It has further been proposed that an increase in cytochrome oxidase gene expression might be the result of an early mitochondrial alteration related to increased oxidative damage (Manczak et al., 2004). In addition to genes involved in OXPHOS subunits, several genes associated with mitochondrial ribosomal protein of the small (two genes) and large (five genes) subunits were also significantly downregulated. It has also been suggested that mitochondrial ribosomal proteins may have a role in cancer, apoptosis and other metabolic diseases (Han, Chiu & Koc, 2010). Moreover, in a recent study, it has been shown that 4 weeks of NP exposure caused mitochondria dysfunction and resulted in the accumulation of reactive oxygen species (ROS) in mouse oocyte (Xu et al., 2020). Taken together, we suggest that adaptive exposure to NP may cause cellular and oxidative stress in yeast cells which increase energy (ATP) demand to overcome the stress.

### NP altered the expression of genes associated with iron and copper acquisition

Iron (Fe) and copper (Cu) are essential cofactors involved in several biochemical reactions, including electron transfer during respiration and protection against oxidative stress (Marvin, Williams & Cashmore, 2003). In the present study, several genes associated with Fe and Cu acquisition were significantly affected upon exposure to NP (Supplemental Materials 2 and 3). Specifically, Fe homeostasis genes *FRE4*, *FRE5* and *FRE8* were downregulated, while *FET3*, *FTR1*, *FTH1* involved in Fe transport across the plasma membrane and into vacuolar; *FIT2*, *FIT3* involved in the retention of siderophore-iron in the cell wall; *SIT1* involved in siderophore iron transport; and *ENB1*, a ferric enterobactin transporting gene, were significantly upregulated in response to NP. It has been previously shown that acute exposure to lower concentrations of NP in medaka fish result in alteration of genes involved in Fe metabolism (Won, Woo & Yum, 2014). Three genes associated with Cu transport, *CTR1* (upregulated), *CTR3* (downregulated) and *CTR86* (downregulated), and two genes involved in CU homeostasis, *REE1* (upregulated) and *ICS3* (downregulated), were also significantly altered after NP exposure. These data suggest that NP can disturb the metal homeostasis in *S. cerevisiae*.

**Figure 6:**
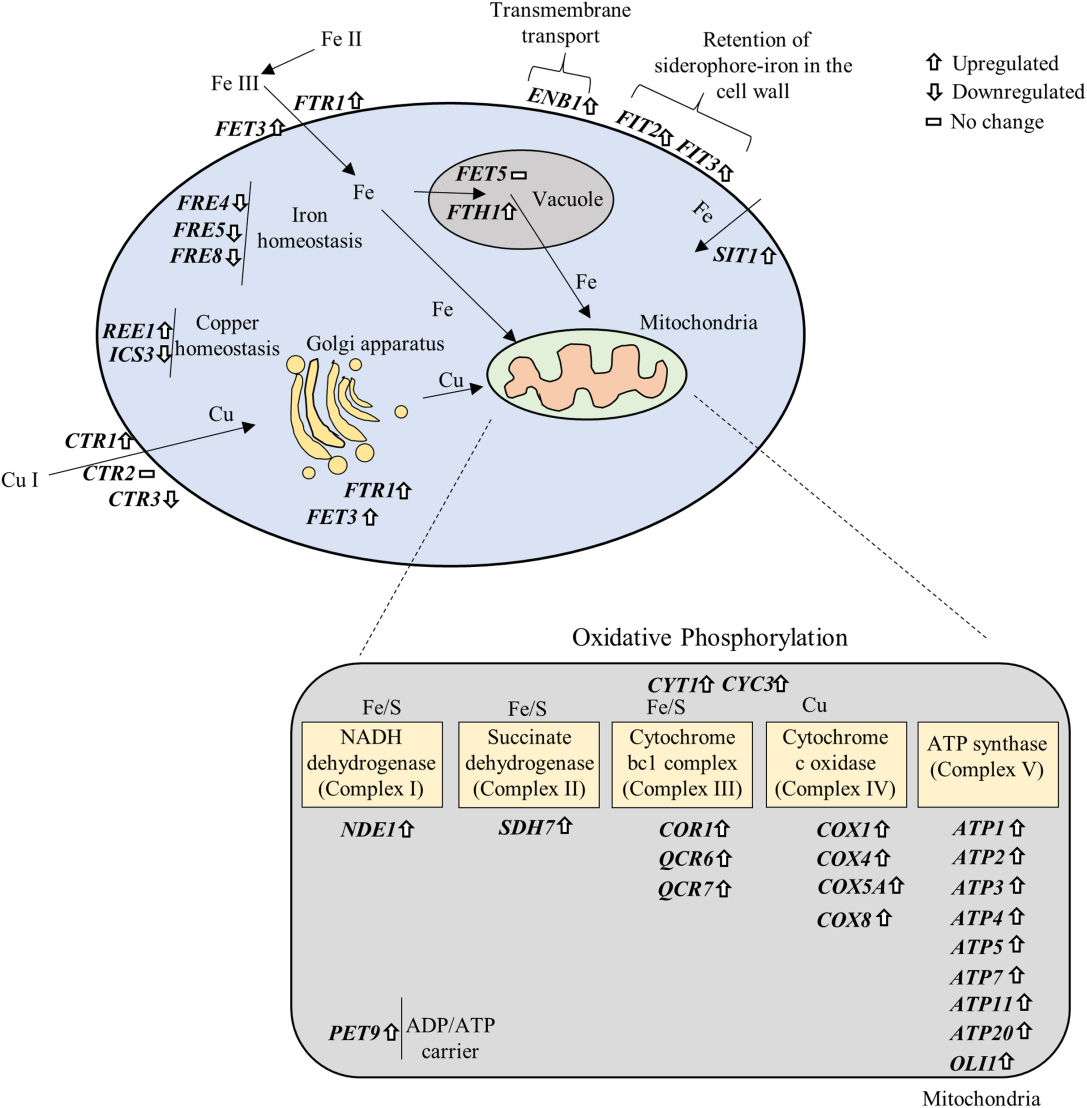
Gene–gene and gene–pathway interactions. The differentially expressed genes were plotted in the iron and copper metabolism and OXPHOS system based on their roles in the pathways.

Fe and Cu are key metals in mitochondrial metabolism, where they play essential roles in the production of ATP (Xu, Barrientos & Andrews, 2013). Fe-S cluster is an essential components of the mitochondrial inner membrane complexes (Complex I, II and III) (Winge, 2012), while Cu is involved in Complex IV (Yoshikawa, Muramoto & Shinzawa-Itoh, 2011) to constitute the electron transport chain. We further prepared a signaling pathway for the above mentioned genes by modifying iron and copper metabolism pathway (De Freitas et al., 2003) and KEGG OXPHOS pathway (Kanehisa & Goto, 2000). The results showed that overexpression of OXPHOS system is mediated by upregulation of iron and copper related genes (Fig. 6).

### NP repressed autophagy-related and pleiotropic drug resistance genes

Autophagy is a highly conserved pathway in eukaryotes in which cytoplasmic contents are transported to lysosomes for degradation and then utilized for protein synthesis or oxidized by mitochondria to produce energy for cell survival during starvation (Chen & Klionsky, 2011). In *S. cerevisiae*, around 30 autophagy-related (Atg) genes are reported and null mutations in these genes can affect autophagy process and this in turn, leads to increased cell death under nutrient starvation conditions (Suzuki & Ohsumi, 2007). In the present study, four Atg genes were significantly downregulated (Supplemental Material 2). Interestingly, this finding contrasts with that of the previous study, which the expression of Atg genes yeast were significantly induced in response to short-term exposure of NP (Bereketoglu et al., 2017b).

Several genes associated with the pleiotropic drug resistance (PDR) network were also altered (Supplemental Material 2). Of these genes, *PDR5* was the only upregulated gene, while the other three genes (*PDR12*, *PDR17* and *PDR18*) were significantly repressed. PDR network is constitutively regulated by the transcription factors Pdr1p and Pdr3p in *S. cerevisiae* (Bauer, Wolfger & Kuchler, 1999). This network is involved in multidrug resistance (MDR) (Bauer, Wolfger & Kuchler, 1999). *PDR5* encodes the multidrug efflux transporter (ATP-binding cassette (ABC) transporter) Pdr5p. Pdr5p is positively regulated by Pdr1p and Pdr3p. It provides resistance to various xenobiotic compounds, including mutagens, fungicides, steroids, and anticancer drugs (Bauer, Wolfger & Kuchler, 1999). The ABC superfamily of proteins is highly conserved from prokaryotes to higher eukaryotes. Moreover, the increased expression of human ABC transporter ABCB1/MDR1 has been observed in tumours that provides resistance to drug therapy and loss of function of this transporter is linked with several human diseases (Bauer, Wolfger & Kuchler, 1999; Decottignies & Goffeau, 1997). Altogether, the present result is consistent based on *PDR5* expression while PDR gene expression was not consistent with our previous studies on NP (Bereketoglu et al., 2017b), BPA (Bereketoglu et al., 2017a) and other toxicity studies on (Nishida et al., 2014), alachlor (Gil et al., 2011) and p-anisaldehyde (Yu et al., 2010).

### NP affected cell cycle progression and related processes/pathways

The eukaryotic cell cycle ensures the maintenance of cell size and the level of ploidy. To enable and control the progression through the cell cycle and nuclear division, a subset of genes is required for regulation of cell cycle progression, maintenance of genome integrity by detecting DNA damage, encoding proteins necessary for DNA repair, arresting cells for mismatch repair, and spindle checkpoint (Azzopardi, Farrugia & Balzan, 2017; Teufel et al., 2019). Several cell cycle progression genes were significantly downregulated in response to NP (Figs. 3 and 4). Of these, *CLN2* encodes a G1 cyclin involved in cell cycle regulation and activates the Cdc28p kinase to promote G1/S phase transition. *CLB2* encode B-type cyclins involved in cell cycle progression that also activates Cdc28p, but promote the G2/M transition (Fitch et al., 1992). In addition, *CLB6* is another B-type cyclin encoding gene that is associated with DNA replication during S phase and promotes initiation of DNA synthesis by activating Cdc28p (Lew, 1997). Our findings contrast with other previous NP exposure studies performed on Atlantic salmon smolts, juvenile rainbow trout and prostate epithelial cells which showed induced expression of cell cycle and differentiation genes (Forte et al., 2016; Robertson & McCormick, 2012; Shelley et al., 2012). Taken together, our findings suggest that NP may inhibit the cell cycle progression in yeast cells.

DNA damage under environmental stresses or cellular processes may cause genomic instability, mutagenesis or even cell death (Fu, Pastushok & Xiao, 2008; Gasch et al., 2001). Therefore, cells have orchestrated complex surveillance pathways to ensure faithful cell division, monitor genomic integrity during normal cell cycle progression and in response to DNA damage agents (Chabes et al., 2003; Gasch et al., 2001; Lao et al., 2018). In the present study, NP repressed the expression of several DNA damage response genes including *RAD* genes, *MEC1* and *DUN1* genes which encode checkpoint proteins that promote cell cycle arrest in response to DNA damage and are required for the repair of double-strand breaks (Gasch et al., 2001; Lao et al., 2018; Schaus, Cavalieri & Myers, 2001). It has been shown that mutations in the above mentioned genes in yeast cells have defects in cell cycle arrest and gene expression responses, and these cells show higher sensitivity to DNA damaging agents (Bashkirov et al., 2000; Desany et al., 1998). In contrast to the present finding, it has been previously demonstrated that acute NP exposure upregulated genes related to DNA damage and apoptosis in juvenile rainbow trout (Shelley et al., 2012). We suggest that this might be due to species differences as well as the exposure period. In addition, we observed decreased expression of genes associated with mismatch repair (MMR) such as *MSH* and *MLH* genes. MMR is a highly conserved mechanism that plays a critical role in faithful replication of genetic material by excising DNA mismatches introduced by DNA polymerase (Chakraborty, Dinh & Alani, 2018). In *S. cerevisiae*, multiple MutS MSH and MLH have evolved to function as heterodimers with specialized functions in DNA repair and recombination (Demogines et al., 2008). Taken together, the present findings suggest that the *S. cerevisiae* show adaptive response towards NP exposure by inhibiting the expression of genes related to DNA and mismatch repair, cellular responses to DNA damage, and cell cycle progression.

Molecular and biochemical analyses have shown that various proteins are required for proper spindle function in cells including microtubules, kinetochores, spindle poles, microtubule based motor proteins and multiple regulatory enzymes (Geiser et al., 1997). We have observed decreased expression of several genes involved in chromosome segregation, spindle checkpoint activity, and kinetochore clustering in response to NP exposure. Particularly, the affected genes encode proteins required for spindle pole body (SPB) insertion and duplication, mitotic nuclear migration, mitotic spindle positioning, kinetochore assembly, clustering and function, and kinetochore-microtubule attachment. In a previous study, it has been indicated that NP exposure disrupted meiotic spindle organization and caused chromosome misalignment in mice (Xu et al., 2020). Besides, several genes associated with telomerase activities such as telomere length regulation, telomere clustering, and capping were significantly downregulated. Telomeres are nucleoprotein structures located at the ends of chromosomes that protect chromosome ends from degradation and deleterious chromosomal rearrangements (Chan & Blackburn, 2002; Collins & Mitchell, 2002; Romano et al., 2013). It has been suggested that environmental stress may affect the regulation and the activity of the genes involved in telomerase activity and consequently perturb telomere length homeostasis (Romano et al., 2013). Taken together, our observations suggest that NP exposure may cause spindle mis-orientation, disrupt kinetochore functions, and impair kinetochore-microtuble attachment, leading to the generation of bi- and multi-nucleated cells.

## Conclusion

In summary, genome-wide transcriptional profiles of *S. cerevisiae* were analyzed to provide useful information for understanding the mode of actions of NP exposure in yeast cells. The present results show that NP affected genes involved in OXPHOS, iron and copper metabolism, autophagy, pleiotropic drug resistance, spindle and kinetochore functions, DNA repair mechanisms, nuclear division, chromosomal segregation, telomerase activity and cytokinesis, indicating that NP exposure triggers mechanisms associated with stress, energy metabolism, cell cycle progression and DNA repair in yeast. In conclusion, the results presented here provide critical insights into the adaptive response of *S. cerevisiae* to NP exposure and the data could be helpful in understanding its molecular mechanisms of action.

## Supplemental Information

10.7717/peerj.10794/supp-1 Supplemental Information 1 Number of dead cells upon exposure to various NP concentrations.

10.7717/peerj.10794/supp-2 Supplemental Information 2 The complete list of differentially expressed genes in *Saccharomyces cerevisiae* exposed to 2 mg/L NP.

10.7717/peerj.10794/supp-3 Supplemental Information 3 Significantly affected GO terms in *Saccharomyces cerevisiae* exposed to 2 mg/L NP.

10.7717/peerj.10794/supp-4 Supplemental Information 4 Significantly affected pathways in *Saccharomyces cerevisiae* exposed to 2 mg/L NP.

10.7717/peerj.10794/supp-5 Supplemental Information 5 Sequences of the primers used for qPCR-based validation of RNASeq data.

10.7717/peerj.10794/supp-6 Supplemental Information 6 Comparison the DEGs of the present study with our previous study (Bereketoglu et al., 2017b). (A) Comparison of 2 mg/L NP adaptive response exposure with 1 and 5 mg/L NP short-term exposure (120 min). (B) Comparison of 2 mg/L NP adaptive response treatment with1 and 5 mg/L mg/L NP short-term (180 min).

10.7717/peerj.10794/supp-7 Supplemental Information 7 Supplemental data for Figure 1.

10.7717/peerj.10794/supp-8 Supplemental Information 8 Supplemental data for Figure 5.

## Additional Information and Declarations

### Data Availability

The following information was supplied regarding data availability: The RNA-Seq data is at NCBI GEO: GSE158124.
