# Supplementary material for: Transcriptomic analysis of nonylphenol effect on Saccharomyces cerevisiae
Source: PeerJ. 2021 Feb 11;9:e10794. doi: 10.7717/peerj.10794 (PMC7882136; doi:10.7717/peerj.10794)
Supplement: Supplemental Information 5 [file peerj-09-10794-s005.docx]

| **Gene symbol** | **Gene name** | **Forward primer (5´–3´)** | **Reverse primer (5´–3´)** |
| --- | --- | --- | --- |
| *QCR7* | *ubiQuinol-cytochrome C oxidoReductase* | GTCCTCTCCAAGTTATGTGTTCCA | CGGTTTGATGAGCCCTGATTATTC |
| *ATP3* | *ATP synthase* | TGACCCAGTGTCTTCCCTATCTTT | CAGCAGCATAACCTTGAGCCATT |
| *COX4* | *Cytochrome c OXidase* | AACCATTAGATTCGTCCAGGAAGG | GTATGTGAACCAGCGGGAGAAC |
| *SOD1* | *SuperOxide Dismutase* | TGCTGGTCCTCACTTCAATC | TTCGTCCGTCTTTACGTTACC |
| *PDR5* | *Pleiotropic Drug Resistance* | TGACGCTTTTGCATCAGTTC | GAGAAAACCGCGACAATGTT |
| *ACT1* | *ACTin* | CTGCCGGTATTGACCAAACT | CGGTGATTTCCTTTTGCATT |
