# Supplementary figures and images for: Transcriptomic analysis of nonylphenol effect on Saccharomyces cerevisiae
Source: PeerJ. 2021 Feb 11;9:e10794. doi: 10.7717/peerj.10794 (PMC7882136; doi:10.7717/peerj.10794)

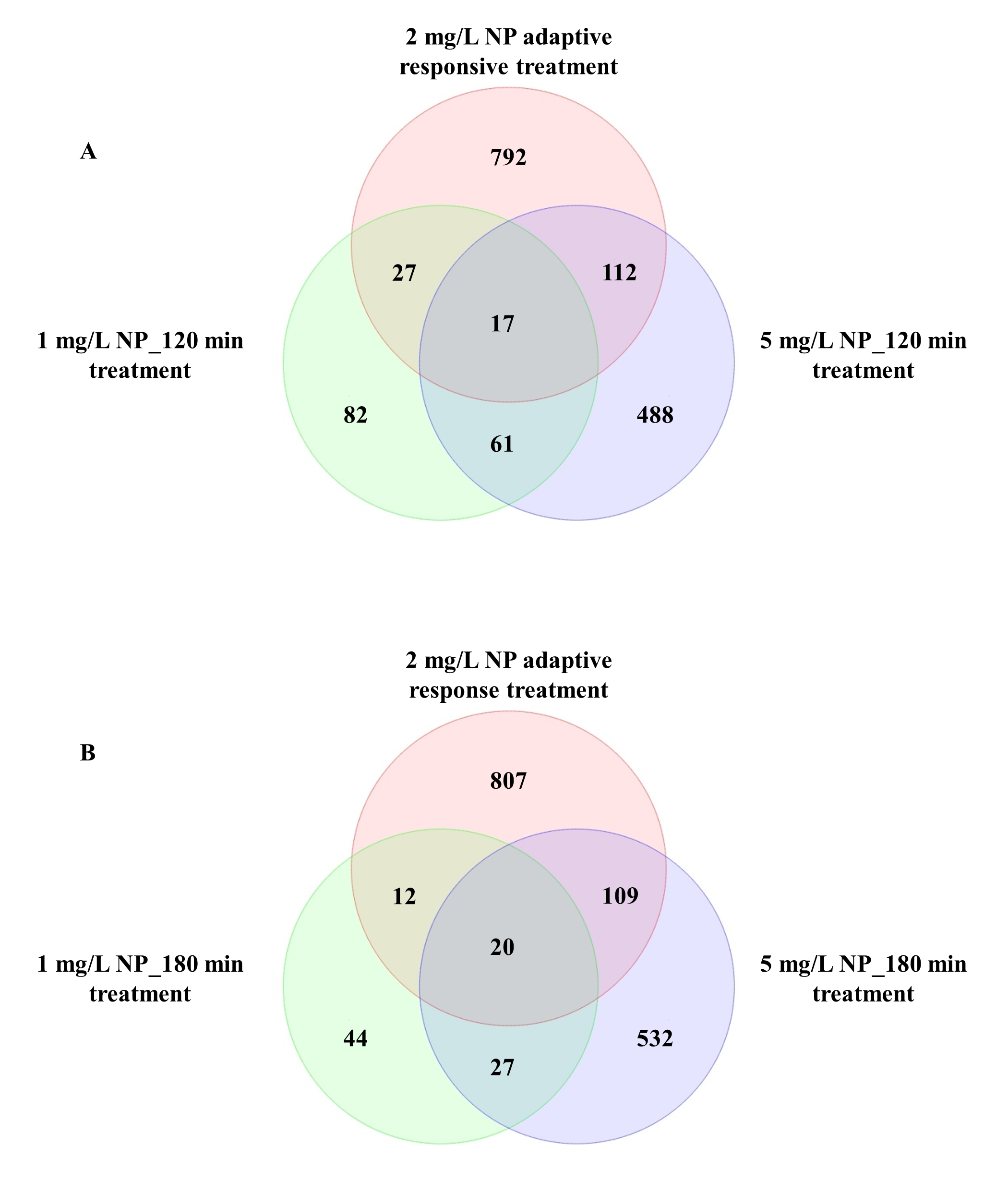

Supplement: Supplemental Information 6 — (A) Comparison of 2 mg/L NP adaptive response exposure with 1 and 5 mg/L NP short-term exposure (120 min). (B) Comparison of 2 mg/L NP adaptive response treatment with1 and 5 mg/L mg/L NP short-term (180 min). [file peerj-09-10794-s006.png]
